# Mutation of the Putative Immunosuppressive Domain of the Retroviral Envelope Glycoprotein Compromises Infectivity

**DOI:** 10.1128/JVI.01152-17

**Published:** 2017-10-13

**Authors:** Urszula Eksmond, Bryony Jenkins, Julia Merkenschlager, Walther Mothes, Jonathan P. Stoye, George Kassiotis

**Affiliations:** a Retroviral Immunology, The Francis Crick Institute, London, United Kingdom; b Retrovirus-Host Interactions, The Francis Crick Institute, London, United Kingdom; c Department of Medicine, Faculty of Medicine, Imperial College London, London, United Kingdom; d Department of Microbial Pathogenesis, Yale University School of Medicine, New Haven, Connecticut, USA; University of Utah

**Keywords:** envelope glycoprotein, immunosuppressive domain, infectivity, murine leukemia virus

## Abstract

The envelope glycoprotein of diverse endogenous and exogenous retroviruses is considered inherently immunosuppressive. Extensive work mapped the immunosuppressive activity to a highly conserved domain, termed the immunosuppressive domain (ISD), in the transmembrane (TM) subunit of the envelope glycoprotein and identified two naturally polymorphic key residues that afford immunosuppressive activity to distinct envelope glycoproteins. Concurrent mutation of these two key residues (E14R and A20F) in the envelope glycoprotein of the Friend murine leukemia virus (F-MLV) ISD has been reported to abolish its immunosuppressive activity, without affecting its fusogenicity, and to weaken the ability of the virus to replicate specifically in immunocompetent hosts. Here, we show that mutation of these key residues did, in fact, result in a substantial loss of F-MLV infectivity, independently of host immunity, challenging whether associations exist between the two. Notably, a loss of infectivity incurred by the F-MLV mutant with the E14R and A20F double ISD mutation was conditional on expression of the ecotropic envelope receptor murine cationic amino acid transporter-1 (mCAT1) in the virus-producing cell. Indeed, the F-MLV mutant retained infectivity when it was produced by human cells, which naturally lack mCAT1 expression, but not by murine cells. Furthermore, mCAT1 overexpression in human cells impaired the infectivity of both the F-MLV double mutant and the wild-type F-MLV strain, suggesting a finely tuned relationship between the levels of mCAT1 in the producer cell and the infectivity of the virions produced. An adverse effect on this relationship, rather than disruption of the putative ISD, is therefore more likely to explain the loss of F-MLV infectivity incurred by mutations in key ISD residues E14 and A20.

**IMPORTANCE** Retroviruses can interact with their hosts in ways that, although not entirely understood, can greatly influence their pathogenic potential. One such example is a putative immunosuppressive activity, which has been mapped to a conserved domain of the retroviral envelope glycoprotein of several exogenous as well as endogenous retroviruses. In this study, mutations naturally found in some envelope glycoproteins lacking immunosuppressive activity were shown to affect retrovirus infectivity only if the host cell that produced the retrovirus also expressed the cellular entry receptor. These findings shed light on a novel role for this conserved domain in providing the necessary stability to the envelope glycoprotein in order to withstand the interaction with the cellular receptor during virus formation. This function of the domain is critical for further elucidation of the mechanism of immunosuppression mediated by the retroviral envelope glycoprotein.

## INTRODUCTION

Retroviruses are enveloped viruses with a genomic structure that includes, in its simplest form, three open reading frames, *gag*, *pro-pol*, and *env*, encoding proteins with structural or enzymatic functions (1, 2). Whereas more complex retroviruses may additionally encode accessory proteins, the common proteins are thought to serve the same function in all retroviruses (3). For example, the major protein on the surface of retroviral particles in all retroviruses is the retroviral envelope glycoprotein, encoded by the *env* (envelope) open reading frame (2, 4). Its main function is binding to the cellular receptor and mediating membrane fusion, thus allowing retroviral entry into the target cell. However, in addition to receptor binding and membrane fusion, the envelope glycoprotein is also implicated in resistance to superinfection (5), as well as immunomodulation (6–11), through its different domains. Indeed, the newly synthesized envelope glycoprotein in the infected cell may also interact with the cellular receptor either in the endoplasmic reticulum or at the cell surface, and this interaction underlies resistance to superinfection with retroviruses using the same cellular receptor (5, 12).

Receptor binding and membrane fusion are mediated by the homotrimeric complex of the envelope glycoprotein on the surface of retroviral particles (13, 14). Each monomer of the complex comprises two subunits, the surface unit (SU) and the transmembrane (TM), which are created following cleavage of the *env*-encoded single polypeptide by a cellular protease in the Golgi apparatus (13). Large and variable numbers of predominantly *N*-linked glycosylation sites are also modified in the SU during translation (13). The SU and TM subunits are typically associated by a noncovalent interaction between the C-terminal domain of the SU and the N-terminal domain of the TM, both of which are enriched in hydrophobic, nonpolar residues (15, 16). The N-terminal domain of the TM also contains the fusion peptide, which mediates membrane fusion (14–16). The C-terminal domain of the Friend murine leukemia virus (F-MLV) SU carries a disulfide bond isomerase motif, CXXC (17), which is bonded to the TM by a disulfide bond (18). Binding of the SU to the cellular receptor leads to reshuffling of the disulfide bonds, creating an intramolecular bridge in the CXXC motif of the SU (19). This reshuffling of the disulfide bridges separates the SU and TM subunits, exposing the fusion peptides in the TM monomers, which then insert into the target cell membrane, thereby initiating the fusion process (19).

Also present in the ectodomain of the TM is a conserved region with a putative immunosuppressive function (8). This immunosuppressive domain (ISD) overlaps with regions of the TM, whose conformational changes are essential for the fusion process, and these constraints may partly account for the conservation of TM in diverse endogenous and exogenous retroviruses (4, 7, 8, 11). Evidence for the immunosuppressive activity of the TM has been provided by multiple experimental systems involving the envelope glycoproteins of several different exogenous as well as endogenous retroviruses (4, 7, 8, 11). Immunosuppressive activity was also demonstrated in human and murine syncytins, endogenous retroviral envelope glycoproteins, independently captured from distinct endogenous retroviruses and exapted in mammalian placentation (8). Interestingly, seminal work by Heidmann and colleagues revealed that only one of the two syncytins retains immunosuppressive activity in both humans and mice, whereas both syncytins are fusogenic (20). Residues R14 and F20 in the TM of the nonimmunosuppressive human Syncytin-1 distinguish it from the TM of immunosuppressive Syncytin-2 and other retroviral envelope glycoproteins (20). Indeed, the E14R and A20F double mutation in the otherwise immunosuppressive TM of F-MLV has been reported to render the virus highly susceptible to immune-mediated elimination (10).

Despite the potential importance of the immunosuppressive activity of the retroviral envelope glycoprotein in tuning immune reactivity to exogenous and endogenous retroviruses, the precise molecular targets of the immunosuppressive domain remain unknown. In our efforts to elucidate the requirements for immunity to F-MLV infection, we reexamined the effects of the E14R and A20F double mutation. In contrast to previous reports, our results indicate that these specific mutations in this region of the TM are, in fact, detrimental to virus infectivity, irrespective of immunogenicity, but are dependent on the presence of the cellular receptor in the producer cell. Therefore, the viral attenuation arising from these two mutations should not be taken as evidence for a lack of immunosuppression.

## RESULTS

### ISD mutants are conditionally replication defective.

**FIG 1:**
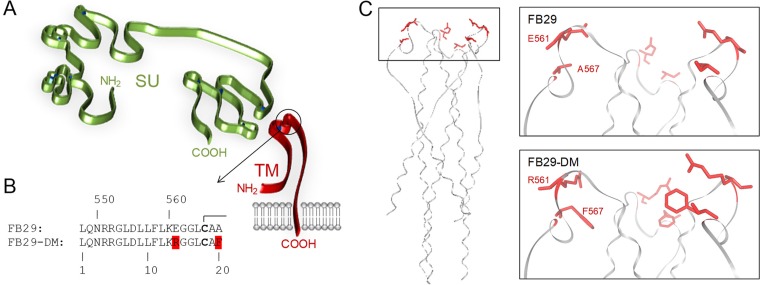
Location of the double mutation in the F-MLV TM in relation to the predicted structural domains of the SU and the TM. (A) Schematic representation of the SU and TM domains, based on models proposed for the MLV SU (15) and the MLV TM (21), respectively. The circled region at the apex of the TM monomer corresponds to the ISD. (B) Sequence of the wild-type and double mutant F-MLV ISD. The numbers on the top row correspond to the amino acid positions in the entire envelope polypeptide, whereas those on the bottom row correspond to the amino acid positions in ISD only. Bold fonts indicate the cysteine residues involved in disulfide bonds. (C) Structure of the MLV TM trimer (Protein Data Bank accession number 4JGS) (21), highlighting the positions of the E561 and A567 residues (left), and structural model of the FB29 (wild type) or the FB29-DM (double mutant) TM apex (right).

To examine the possible immune effects of mutations in the putative ISD of the F-MLV envelope, we generated a previously reported mutant in which fusogenicity was retained while immunosuppression was abolished (10). The key residues E561 and A567 in the envelope of F-MLV clone FB29 were replaced with R561 and F567, respectively (E14R and A20F, respectively, in the ISD) (Fig. 1A and B), which are naturally found in nonimmunosuppressive human Syncytin-1 (10, 20). This double mutant is referred to here as FB29-DM, for consistency with the published literature (10). Inspection of the 3-dimensional structure of the murine leukemia virus (MLV) TM (21) indicated that these two amino acids are located at the apex of each of the three TM monomers in the mature envelope glycoprotein (Fig. 1C). Further modeling suggested that the surface created by these amino acids at the apex of the TM is likely to be altered by the double E14R and A20F mutation (Fig. 1C).

**FIG 2:**
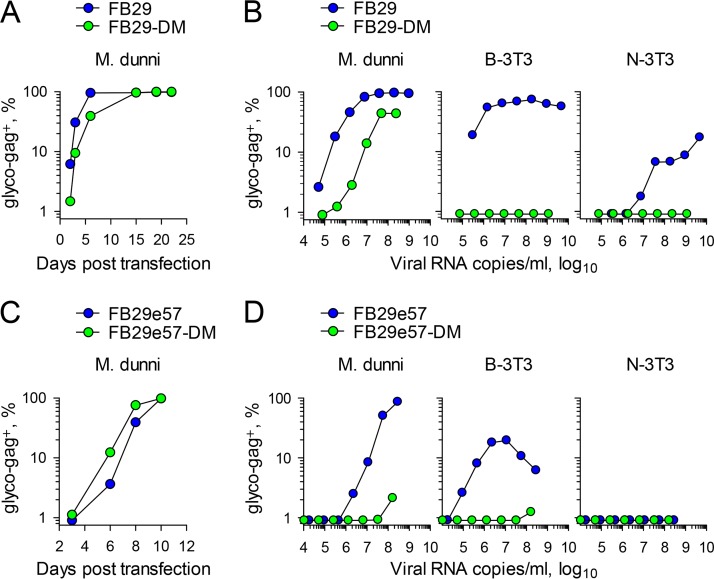
Infectivity of wild-type and ISD mutant F-MLVs produced by murine cells. (A) Frequency of M. dunni cells positive for F-MLV glyco-Gag over time after transfection with plasmids containing the FB29 (wild-type) or the FB29-DM (E14R and A20F double mutant) genome. The results of one representative of two experiments conducted are shown. (B) Frequency of F-MLV-infected (glyco-Gag-positive [glyco-gag^+^]) M. dunni, B-3T3, or N-3T3 cells 3 days after infection with the indicated doses of FB29 or FB29-DM viruses produced by transfection of M. dunni cells. The results of one representative of three experiments conducted are shown. (C) Frequency of M. dunni cells positive for F-MLV glyco-Gag over time after transfection with plasmids containing the FB29e57 or FB29e57-DM genome. The results of one representative of two experiments conducted are shown. (D) Frequency of F-MLV-infected (glyco-Gag-positive) M. dunni, B-3T3, or N-3T3 cells 3 days after infection with the indicated doses of FB29e57 or FB29e57-DM viruses produced by transfection of M. dunni cells. The results of one representative of three experiments conducted are shown.

Plasmids containing the FB29 or FB29-DM genome were transfected into Mus dunni cells, and virus production and spread were monitored by staining for the F-MLV glycosylated Gag (glyco-Gag). Both cultures became uniformly positive for F-MLV glyco-Gag within 10 days (Fig. 2A), indicating efficient spread of the FB29 and FB29-DM viruses.

To confirm that the FB29-DM variant retained full infectivity potential *in vitro*, we additionally tested its ability to acutely infect new M. dunni cells. Serial dilutions of supernatants from chronically infected M. dunni cells were transferred onto new M. dunni cells, which were tested for glyco-Gag expression 3 days later. Surprisingly, although they contained comparable numbers of F-MLV RNA copies per volume unit, supernatants from FB29-DM-producing M. dunni cells were ∼100 times less infectious than those from wild-type FB29-producing M. dunni cells (Fig. 2B). The same supernatants were additionally tested on BALB/c mouse 3T3 (B-3T3) cells and NIH 3T3 (N-3T3) cells. The FB29 genome encodes an NB-tropic capsid and is therefore capable of infecting both B-3T3 and N-3T3 cells (22, 23). Indeed, both cell types were infected by supernatants from FB29-producing M. dunni cells, although N-3T3 cells were infected with much less efficiency (Fig. 2B), likely due to their reduced expression of murine cationic amino acid transporter-1 (mCAT1) (24, 25), the cellular receptor for ecotropic retroviral envelope glycoproteins (26). In stark contrast, supernatants from M. dunni cells producing FB29-DM were completely unable to infect either B-3T3 or N-3T3 cells (Fig. 2B).

The apparent loss of infectivity of M. dunni cell-produced FB29-DM was at odds with a published report in which the E14R and A20F double mutation was introduced into the envelope of F-MLV clone 57 without an apparent loss of infectivity (10). The envelopes of F-MLV clones FB29 and 57 are highly similar (96.8% amino acid identity) (27) but not identical. To exclude the possibility that the discordant results were due to the use of different F-MLV envelopes, we replaced the envelope of clone FB29 with either the envelope of wild-type clone 57 (FB29e57) or the envelope of clone 57 carrying the E14R and A20F double mutation (FB29e57-DM). M. dunni cells transfected with plasmids containing the FB29e57 or FB29e57-DM genome became uniformly infected within 10 days, reflected in their glyco-Gag expression (Fig. 2C). However, when supernatants from M. dunni cells were added to new M. dunni cells, supernatants from FB29e57-DM-producing M. dunni cells were >100 times less infectious than those from wild-type FB29e57-producing M. dunni cells (Fig. 2D). Moreover, wild-type FB29e57 but not FB29e57-DM produced by M. dunni cells was able to infect B-3T3 cells, and at higher titers, it caused cytopathic effects, resulting in an apparent drop in infectivity, whereas neither virus could infect N-3T3 cells (Fig. 2D), again likely due to the reduced expression of mCAT1 in N-3T3 cells (24, 25). Viruses with the wild-type clone 57 envelope were 10 to 100 times less infectious in all three cell lines than those with the wild-type clone FB29 envelope. Nevertheless, the E14R and A20F double mutation in both envelopes appeared to considerably reduce infectivity (in M. dunni cells) or abolish infectivity (in B-3T3 and N-3T3 cells).

**FIG 3:**
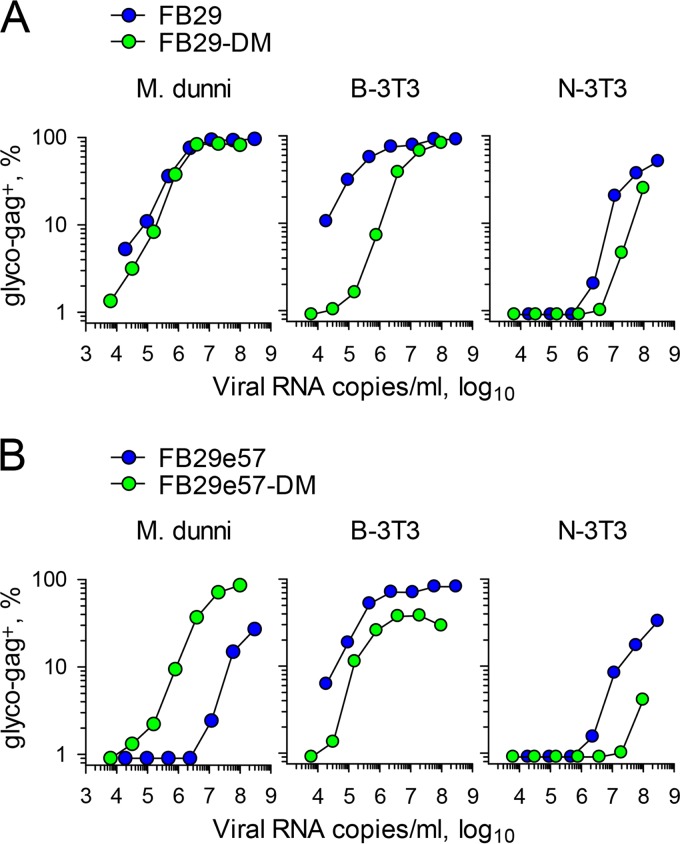
Infectivity of wild-type and ISD mutant F-MLVs produced by human cells. (A) Frequency of F-MLV-infected (glyco-Gag-positive) M. dunni, B-3T3, or N-3T3 cells 3 days after infection with the indicated doses of FB29 or FB29-DM viruses produced by transfection of 293T cells. The results of one representative of two experiments conducted are shown. (B) Frequency of F-MLV-infected (glyco-Gag-positive) M. dunni, B-3T3, or N-3T3 cells 3 days after infection with the indicated doses of FB29e57 or FB29e57-DM viruses produced by transfection of 293T cells. The results of one representative of two experiments conducted are shown.

One notable difference between earlier work describing the E14R and A20F double mutation (10) and the work described here is the choice of virus producer cell. Retroviral stocks are often prepared by transfection of human HEK 293 cells (10), whereas we used murine M. dunni cells. To examine if the nature of the producer cell type could account for the discordant results, we produced FB29 and FB29-DM viruses by transfection of 293T cells and tested their infectivity for murine M. dunni, B-3T3, and N-3T3 cells (Fig. 3A). Surprisingly, when virus was produced by 293T cells, the infectivity of FB29-DM for M. dunni target cells was comparable to that of FB29 (Fig. 3A). Moreover, FB29-DM could now also infect B-3T3 and N-3T3 target cells, albeit with an efficiency somewhat reduced from that of FB29 (Fig. 3A). Similar results were also obtained when the FB29e57 and FB29e57-DM viruses were produced by transfection of 293T cells (Fig. 3B). One exception was that the use of the wild-type clone 57 envelope but not the mutated clone 57 envelope reduced the infectivity of the resulting viruses in comparison with that when the wild-type FB29 envelope was used, specifically in M. dunni cells. This finding is likely related to the expression of the mCAT1 variant in these cells, which permits infection by some ecotropic MLV envelopes but not others (28, 29). Of note, whereas both the FB29e57 and FB29e57-DM viruses produced by M. dunni cells could not infect N-3T3 target cells, those produced in 293T cells could (Fig. 3B). Taken together, these results imply that mutations in the ISD of the F-MLV envelope strongly affect virus infectivity if the virus is produced by murine but not human cells.

### ISD mutants fail to spread *in vitro* or *in vivo* independently of immunity.

**FIG 4:**
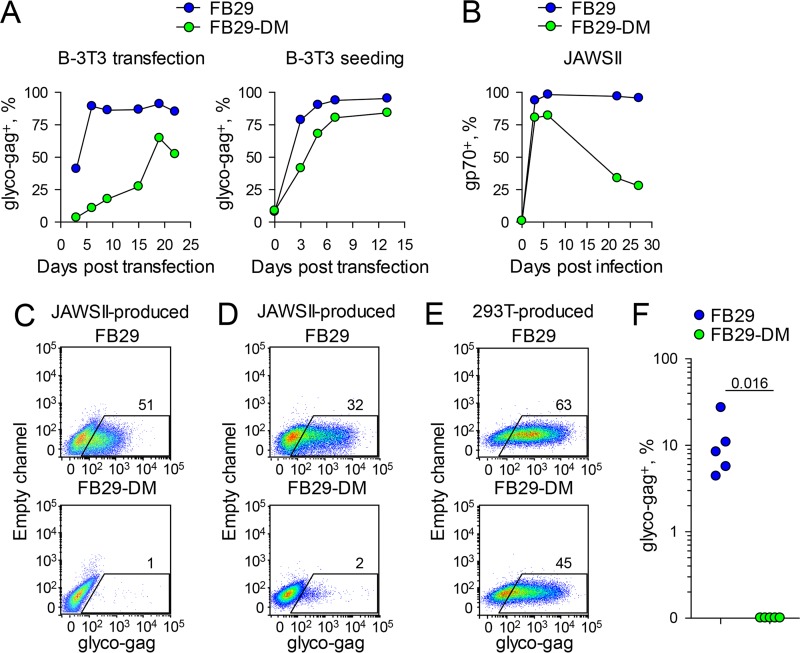
Infectivity of wild-type and ISD mutant F-MLVs in primary murine cells and *in vivo*. (A) (Left) Frequency of B-3T3 cells positive for F-MLV glyco-Gag over time after transfection with plasmids containing the FB29 or FB29-DM genome. (Right) Frequency of B-3T3 cells positive for F-MLV glyco-Gag over time in cultures of FB29- or FB29-DM-infected B-3T3 cells mixed with uninfected B-3T3 cells (at a ratio of 1 infected cell to 9 uninfected cells). The results of one representative of two experiments conducted are shown. (B) Frequency of F-MLV-infected (gp70-positive [gp70^+^]) JAWSII cells over time after infection with FB29 or FB29-DM viruses produced by transfection of 293T cells. (C) Flow cytometric detection of F-MLV-infected (glyco-Gag-positive) JAWSII cells 3 days after infection with FB29 or FB29-DM viruses produced by JAWSII cells. The plots are representative of those from two independent experiments. (D) Flow cytometric detection of F-MLV-infected (glyco-Gag-positive) LPS-stimulated primary B cells 3 days after infection with FB29 or FB29-DM viruses produced by JAWSII cells. The plots are representative of those from two independent experiments. (E) Flow cytometric detection of F-MLV-infected (glyco-Gag-positive) LPS-stimulated primary B cells 3 days after infection with FB29 or FB29-DM viruses produced by 293T cells. The plots are representative of those from two independent experiments. (F) Frequency of F-MLV-infected (glyco-Gag-positive) cells in Ter119-positive erythroid precursors in the spleens of *Rag1*^−/−^ *Il15ra*^−/−^ *Emv2*^−/−^ mice 14 days after intravenous injection of FB29 or FB29-DM viruses produced by M. dunni cells. Each symbol represents an individual mouse. The results of one representative of two experiments conducted are shown.

An obvious difference between human 293T and murine M. dunni virus-producing cells is the presence of the receptor mCAT1 only in the latter. M. dunni cells express an allelic variant of the M. musculus *Slc7a1* gene (encoding mCAT1) compared to the gene found in laboratory mice and B-3T3 and N-3T3 cells (28, 29). The M. dunni protein variant is distinguished by 3 amino acid substitutions and 1 insertion out of 622 amino acid residues in the M. musculus protein, and these changes alter the binding of certain ecotropic envelope glycoproteins (28, 29). It was therefore possible that F-MLV growth in M. dunni cells selected for virus envelope mutants that were adapted to the M. dunni mCAT1 variant and, as a result, were unable to use the M. musculus mCAT1. Sequencing of the entire *env* open reading frame of the FB29 and FB29-DM viruses produced by M. dunni cells revealed no additional mutations (data not shown). Nevertheless, we also examined the ability of FB29 and FB29-DM viruses to replicate in B-3T3 cells. Following transfection with plasmids containing the respective proviral genomes, B-3T3 cells quickly became uniformly infected by FB29 (Fig. 4A). In contrast, the spread of FB29-DM in B-3T3 cells was considerably slower and the virus never reached all of the cells in the culture (Fig. 4A). To control for differences in the efficiency of initial transfection, the replication of FB29 and FB29-DM viruses in B-3T3 cells was also tested in cultures in which the starting frequency of infected B-3T3 cells was normalized by addition of uninfected B-3T3 cells to achieve a ratio of 1 infected cell to 9 uninfected cells. Again, even though both cultures started with the same frequency of infected cells (∼10%), by day 3, cultures seeded with FB29-infected B-3T3 cells contained twice as many infected cells as those seeded with FB29-DM-infected B-3T3 cells (Fig. 4A).

The ability of the FB29 and FB29-DM viruses to replicate in M. musculus cells was additionally tested in JAWSII cells. JAWSII is an immature dendritic cell line derived from C57BL/6 mice. The FB29 and FB29-DM viruses produced in 293T cells were used to infect JAWSII cells, achieving ∼80% infection (Fig. 4B). Whereas in the case of FB29 the proportion of F-MLV-infected JAWSII cells rose to and remained at ∼100%, in the case of FB29-DM, this proportion eventually dropped to <30% (Fig. 4B), likely by the outgrowth of cells that remained uninfected, indicating the defective spread of FB29-DM in JAWSII cell cultures. Moreover, in contrast to FB29, the FB29-DM produced by these JAWSII cells was completely unable to infect new JAWSII cells (Fig. 4C). The loss of infectivity of FB29-DM, when it was produced by murine cells, was not restricted to *in vitro*-grown cell lines, as JAWSII cell-produced FB29-DM was unable to infect lipopolysaccharide (LPS)-activated primary B cells, whereas JAWSII cell-produced FB29 efficiently infected these target cells (Fig. 4D). In contrast to the difference in infectivity when they were produced by JAWSII cells, both the FB29 and FB29-DM produced by 293T cells infected LPS-activated primary B cells with comparable efficiencies (Fig. 4E). These results argue that the loss of FB29-DM infectivity was the result of production by murine cells and was not due to adaptation to the M. dunni mCAT1 variant.

We next examined if the inability of FB29-DM to spread in murine cells was also apparent *in vivo*, where different modes of cell-to-cell transmission may be operating (30). The E14R and A20F double mutation in the F-MLV envelope was previously suggested to render the virus susceptible to immune control, mediated by T cells and NK cells (10). We therefore tested the replication of either the FB29 or FB29-DM produced by M. dunni cells in mice that were T cell, B cell, and NK cell deficient due to a combined deficiency in Rag1 (which is necessary for T cell and B cell development) and the interleukin-15 (IL-15) receptor α chain (which is necessary for NK cell development). These *Rag1*^−/−^ *Il15ra*^−/−^ mice were additionally rendered deficient in *Emv2*, the single ecotropic MLV provirus present in the genome of B6 mice. *Emv2* was removed to prevent ecotropic receptor interference (5), as well as the generation of recombinant infectious MLVs, rescuing potential replication defects (31). Consistent with the *in vitro* and *ex vivo* findings, FB29 but not FB29-DM was able to spread in severely immunodeficient *Rag1*^−/−^ *Il15ra*^−/−^ *Emv2*^−/−^ mice (Fig. 4F), indicating that the lack of FB29-DM infectivity *in vivo* was not due to immune control.

### Defective cell membrane expression and virion incorporation of ISD mutant envelope.

**FIG 5:**
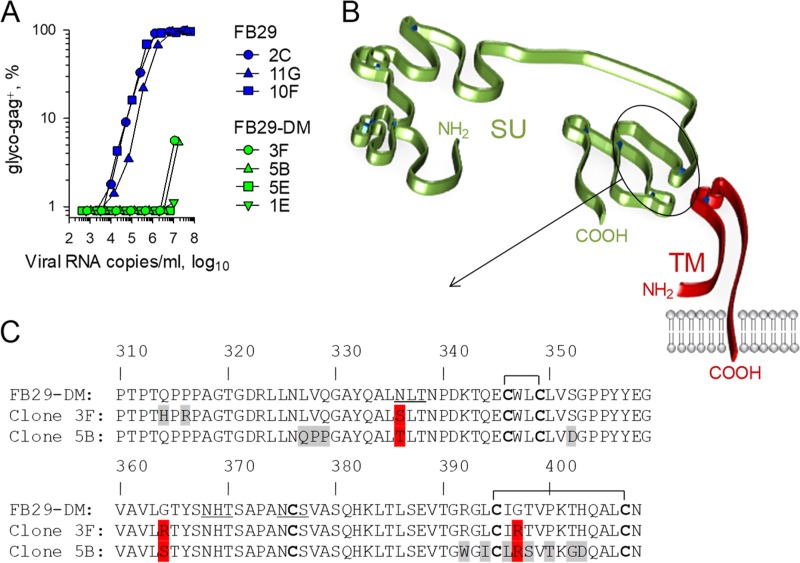
Infectivity of wild-type and ISD mutant F-MLV clones isolated in JAWSII cell sublines. (A) Frequency of F-MLV-infected (glyco-Gag-positive) M. dunni cells 3 days after infection with the indicated doses of FB29 or FB29-DM viruses produced by the respective JAWSII cell sublines. The results of one representative of two experiments conducted are shown. (B) Schematic representation of the SU and TM domains, based on models proposed for the MLV SU (15) and the MLV TM (21), respectively. The circled region of the SU domain indicates the location where the mutations in clones 3F and 5B of the FB29-DM virus were identified. (C) Section of the amino acid sequence of the envelopes of the index isolate and clones 3F and 5B of the FB29-DM virus. Amino acid positions in gray and red represent unique and common substitutions, respectively. Horizontal brackets indicate the cysteine residues involved in disulfide bonds. Underlined residues denote the *N*-linked glycosylation sites.

The apparent replication defects of F-MLV with the E14R and A20F double envelope mutation observed in our study suggested the impairment of envelope function. Such impairment would put the virus under selection pressure to regain envelope function by either reversion to the wild-type sequence or the acquisition of compensatory mutations in other parts of envelope. To investigate this further, we took advantage of the limited replication of FB29-DM in JAWSII cells (Fig. 4B). JAWSII cells were infected with 293T cell-produced FB29 or FB29-DM viruses, and individual cells were cloned 2 weeks later and expanded as separate sublines. When they were tested on new JAWSII cells, the supernatants from three FB29-infected sublines were comparably infectious (Fig. 5A). By comparison, the supernatants from two of four FB29-DM-infected sublines (sublines 1E and 5E) completely lacked infectivity, whereas those from the other two (sublines 3F and 5B) partially regained infectivity (Fig. 5A). To examine if the apparent differences in infectivity were associated with additional mutations, we sequenced the entire *env* open reading frame of the proviruses integrated in these sublines. The sequences obtained confirmed the presence of a single provirus in each subline, validating the cloning approach. All four clones from the FB29-DM sublines had retained the E14R and A20F double mutation (data not shown), likely because only rare combinations in both these positions can preserve the fusogenic potential (10). Notably, in contrast to M. dunni cell-grown viruses, which did not acquire additional mutations, the more severe impact of the double mutation on virus infectivity in JAWSII cells appeared to select for additional mutations. Indeed, whereas FB29-DM clones 5E and 1E, which showed no infectivity, had not acquired any mutations, the partial gain of infectivity of FB29-DM clones 3F and 5B was associated with additional mutations at the hydrophobic, nonpolar C terminus of the SU (Fig. 5B and C), which is thought to participate in the noncovalent association with a similarly hydrophobic, nonpolar region at the N terminus of the TM (16). FB29-DM clones 3F and 5B bore a number of unique nonsynonymous substitutions, suggesting that they were acquired separately (Fig. 5C). They also showed nonsynonymous changes at shared positions, including an *N*-linked glycosylation site at position 336 and, interestingly, the replacement of two hydrophobic, nonpolar glycine residues at positions 364 and 397 with hydrophilic, polar arginine or serine residues (Fig. 5C). Thus, the association between the gain of infectivity, albeit limited, of FB29-DM clones 3F and 5B and substitutions in the region of the SU interacting with the TM suggested that these substitutions may partially compensate for the disabling effect of the E14R and A20F double mutation.

**FIG 6:**
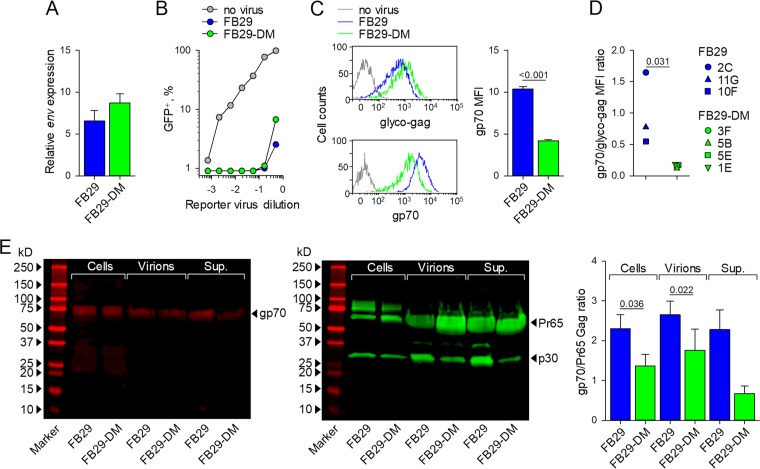
Envelope expression and virion incorporation in wild-type and ISD mutant F-MLVs. (A) Expression of *env* mRNA relative to that of *Hprt* mRNA in M. dunni cells chronically infected with FB29 or FB29-DM viruses following transfection with plasmids containing the FB29 or FB29-DM genome, as in Fig. 2A. Shown are the means ± SEMs (*n* = 3). (B) Frequency of GFP-expressing control M. dunni cells or M. dunni cells chronically infected with FB29 or FB29-DM viruses 3 days after transduction with the indicated doses of an F-MLV envelope-pseudotyped GFP-expressing reporter virus. The results of one representative of two experiments conducted are shown. (C) Flow cytometric detection of F-MLV glyco-Gag or gp70 cell surface expression (left) and median fluorescent intensity (MFI) (means ± SEMs [*n* = 4]) of F-MLV gp70 cell surface staining (right) in uninfected control M. dunni cells or M. dunni cells chronically infected with FB29 or FB29-DM viruses. (D) Ratio of F-MLV gp70 and glyco-Gag cell surface expression (assessed by flow cytometry) in the respective FB29- or FB29-DM-infected JAWSII cell sublines. (E) Western blot analysis of the gp70 (left) and Pr65 Gag and p30 (middle) present in M. dunni cells chronically infected with FB29 or FB29-DM viruses (Cells), the FB29 or FB29-DM virions produced by these cells (Virions), or the total supernatant of these M. dunni cell cultures (Sup.). (Right) Also shown are the ratios of the densitometric values of gp70 and Pr65 Gag in the same samples. The results are representative of those from four experiments conducted (Western blots) or pooled from four experiments (gp70/Pr65 Gag ratio).

We next examined which particular step in virion formation that could account for the loss of infectivity might be affected by the E14R and A20F double mutation. To this end, we used M. dunni cells that had been transfected with plasmids containing the FB29 or FB29-DM proviral genome and had become uniformly positive for the F-MLV glyco-Gag (Fig. 2A). The levels of transcripts of the F-MLV *env* open reading frame were marginally higher in M. dunni cells infected with FB29-DM than in those infected with FB29 (Fig. 6A). Similarly, M. dunni cells infected with either FB29 or FB29-DM were comparably resistant to superinfection with an F-MLV envelope-pseudotyped green fluorescent protein (GFP)-expressing vector (Fig. 6B). These results suggest that both the FB29 and FB29-DM envelopes were transcriptionally expressed at comparable levels and bound mCAT1 with an affinity sufficient to prevent superinfection. However, when the levels of the F-MLV envelope on the cell surface were examined, we noted a significant defect of the FB29-DM envelope (Fig. 6C). Whereas the levels of F-MLV glyco-Gag were marginally higher in M. dunni cells infected with FB29-DM, the levels of the F-MLV SU (gp70) were significantly lower in M. dunni cells infected with FB29-DM than in those infected with FB29 (Fig. 6C). These findings suggest that the E14R and A20F double mutation reduced cell surface envelope accumulation. Consistent with the findings for M. dunni cells, the ratio of F-MLV SU (gp70) to glyco-Gag was significantly reduced on all sublines of JAWSII cells infected with FB29-DM compared with those infected with FB29 (Fig. 6D), again confirming that for the same amount of glyco-Gag, less SU (gp70) is expressed at the cell surface when the E14R and A20F double mutation is introduced.

In agreement with the reduced cell surface expression assessed by flow cytometry, the levels of mutant gp70 were similarly reduced in comparison with those of wild-type gp70 when they were assessed by Western blotting of whole-cell lysates of FB29- and FB29-DM-infected M. dunni cells, as well as in the virions produced by these cells (Fig. 6E). Incorporation of F-MLV TM in the same virions could not be measured, as the E14R and A20F double mutation abolished binding of the available anti-TM monoclonal antibody, 42/114 (data not shown), consistent with the conformational dependence of the 42/114 epitope (32). A compensatory increase in the levels of soluble gp70 in the supernatant of FB29-DM-infected M. dunni cell cultures, which would suggest enhanced shedding of the SU from the cell surface, was not observed (Fig. 6E). Together, these results indicate a reduced overall stability of the FB29-DM envelope, which in turn reduces its availability in the cell and, subsequently, its incorporation into virions.

### mCAT1 overexpression attenuates wild-type F-MLV infectivity.

**FIG 7:**
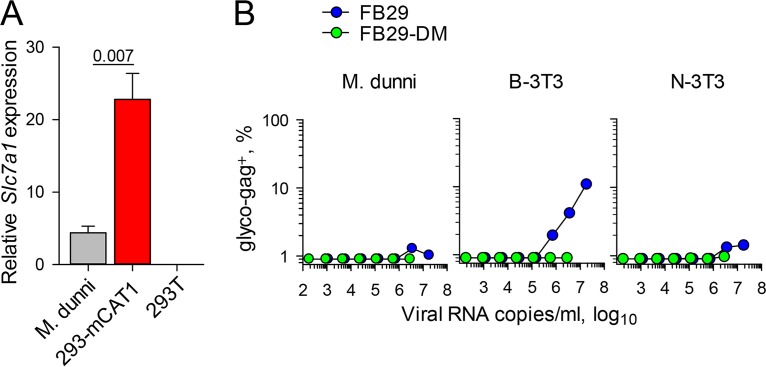
Overexpression of mCAT1 in the producer cell impairs F-MLV infectivity. (A) Expression of *Slc7a1* mRNA (encoding mCAT1) in M. dunni cells, 293T cells, and 293 cells stably transfected with murine *Slc7a1* (293-mCAT1 cells) relative to that of murine *Hprt* or human *HPRT* mRNA in M. dunni and 293 cells, respectively. Shown are the means ± SEMs (*n* = 3). (B) Frequency of F-MLV-infected (glyco-Gag-positive) M. dunni, B-3T3, or N-3T3 cells 3 days after infection with the indicated doses of FB29 or FB29-DM viruses produced by transfection of 293-mCAT1 cells. The results of one representative of three experiments conducted are shown.

The E14R and A20F double mutation seemed to reduce the infectivity of F-MLV virions primarily when they were produced by murine cells. This double mutation did not affect mCAT1 sequestration and, consequently, resistance to superinfection, but it did affect envelope stability and availability. We therefore hypothesized that the E14R and A20F double mutation altered the interaction of the F-MLV envelope with its cellular receptor, mCAT1, in a way that damaged the envelope or led to its degradation. This hypothesis would be entirely consistent with the lack of an effect of the E14R and A20F double mutation on the infectivity of F-MLV virions produced by human cells, which naturally lack mCAT1. To further test if the level of expression of mCAT1 was sufficient to impair the infectivity of F-MLV with the double mutation, we used 293 cells that had been stably transfected with *Slc7a1* (encoding mCAT1) (293-mCAT1 cells) (33). The level of expression of *Slc7a1* in 293-mCAT1 cells was approximately 5 times higher than that in M. dunni cells (Fig. 7A). 293-mCAT1 cells were transfected with plasmids containing the FB29 or FB29-DM genome, and the infectivity of the virus produced was tested on murine cells (Fig. 7B). In stark contrast to the full infectivity of the viruses produced by 293 cells (Fig. 3A), the infectivity of FB29-DM produced by 293-mCAT1 cells was severely reduced or completely abolished (Fig. 7B). Surprisingly, overexpression of mCAT1 in 293 cells also affected wild-type FB29 (compare Fig. 3A and 7B). Thus, overexpression of mCAT1 in human cells was sufficient to abolish the infectivity of the FB29-DM virions produced by these cells and also reduced the infectivity of wild-type FB29 virions.

## DISCUSSION

The retroviral envelope glycoprotein is tasked with distinct functions carried out by well-defined domains (4, 13, 14). In addition to activity involving attachment to the cellular receptor, membrane fusion during viral entry, and resistance to superinfection of already infected cells, the retroviral envelope glycoprotein is also thought to possess immunosuppressive activity (7, 8). This activity has been mapped to a conserved domain of the TM subunit (34), which also participates in the conformational changes that occur during membrane fusion. The R14 and F20 amino acid residues naturally found in the ISD of human Syncytin-1 abolish its immunosuppressive activity, without affecting its fusogenic potential (20). Introduction of these residues (the E14R and A20F double mutation) into the normally immunosuppressive TM subunit of F-MLV was previously reported to render this virus unable to replicate in immunocompetent mice (10), suggesting that this double mutation inactivates a virulence factor that allows it to escape immune detection. The findings that we report here confirm the essential requirement for residues E14 and A20 in the ISD of F-MLV for its replication (10). However, our results further suggest that the E14R and A20F double mutation impacts infectivity independently of immune-mediated control. Indeed, the F-MLV mutant was greatly impaired in its ability to replicate in murine cells both *in vitro* and in severely immunodeficient mice.

Whether or not the E14R and A20F double mutation also affects the immunosuppression exerted by the F-MLV envelope glycoprotein, which may contribute to its virulence, particularly in immunocompetent mice (10), was not addressed in our study. The potential for the F-MLV envelope glycoprotein to alter immune reactivity also was not directly examined in our study. Inoculation of immunocompetent mice with F-MLV with the E14R and A20F double mutation failed to elicit an adaptive immune response (data not shown), in agreement with published data (10). However, we interpreted this lack of immunogenicity of the F-MLV mutant to be a direct consequence of its inability to replicate in immunocompetent mice, also consistent with the findings of the previous study (10). A lack of replication could mask any gain in immunogenicity due to mutations in the putative immunosuppressive domain. Therefore, whether retroviral envelope glycoproteins have immunosuppressive potential and whether the mechanism of immunosuppression is mediated by retroviral envelope glycoproteins remain open questions.

The immunosuppressive potential of the retroviral envelope glycoprotein notwithstanding, our findings are more consistent with the notion that the inability of F-MLV with the E14R and A20F double mutation to replicate is not due to the immune response. Instead, this is likely the result of an inherent loss of infectivity, which is due to defects in envelope glycoprotein stability. In certain combinations of retrovirus-producing and target cell types, the loss of infectivity incurred by the E14R and A20F double mutant is very substantial, if not complete, raising the question of why it has not been previously observed. It should be noted that this loss of infectivity is dependent to a great extent on the expression of the cellular receptor mCAT1 in the retrovirus-producing cell. Indeed, the E14R and A20F double mutation had no measureable effect on F-MLV infectivity if the retrovirus was produced in human cells, which naturally lack mCAT1 expression and which are typically used to prepare retroviral stocks. Furthermore, F-MLV with the E14R and A20F double mutation could still spread in M. dunni cell cultures, when it was introduced by transfection of its proviral DNA, and could also infect M. dunni cells, albeit with a reduced efficiency. By comparison, the infectivity defect of F-MLV with the E14R and A20F double mutation was much more pronounced when laboratory mouse B-3T3 or JAWSII cells were used *in vitro* or, indeed, in primary lymphocytes *ex vivo* and in immunodeficient mice *in vivo*. Together, these results indicate that the degree of phenotypic attenuation provided by the E14R and A20F double mutation in F-MLV is proportional to the levels of mCAT1 expression in the producer cell and is naturally further modified by the levels of mCAT1 expression in the target cell.

The efficient replication of F-MLV with the E14R and A20F double mutation in murine cell lines or mice rendered immunodeficient (10) could also be due to the acquisition of compensatory mutations. Indeed, the limited replication of mutant F-MLV in JAWSII cells gave rise to an additional mutation in the *env* open reading frame that increased infectivity. The function of the E14R and A20F double mutant envelope glycoprotein may also be restored by phenotypic mixing or recombination with endogenous MLVs, which are typically induced in immunodeficient or irradiated mice (31). To prevent either phenotypic mixing or recombination, we used as hosts immunodeficient mice additionally lacking *Emv2*, encoding the endogenous ecotropic MLV provirus, or any infectious ecotropic MLVs of endogenous origin.

In conclusion, our results strongly support a contribution of the E14 and A20 residues in the F-MLV envelope glycoprotein and, by extension, in that of other retroviruses to overcoming a potential loss of infectivity due to interaction with the cellular receptor in the producer cell. Escape from their cellular receptor appears to be a general problem of enveloped viruses, which bud from the plasma membrane. Different viruses may have solved this problem in different ways. A well-recognized example is the neuraminidase of influenza viruses, which enzymatically cleave the sialic acid groups present in glycoproteins at the plasma membrane of the producer cell (35, 36). These sialic acid groups are the attachment targets of the viral hemagglutinin, and their removal by neuraminidase is essential for virus release (35, 36).

In addition to virus release, interaction of the retroviral envelope glycoprotein with its cellular receptor in the virus-producing cell may have major consequences for the immunogenicity of the virus or its sensitivity to immune effector molecules. For example, the antibodies that are most effective at mediating antibody-dependent cell-mediated cytotoxicity (ADCC) against HIV-1-infected cells target epitopes that are exposed only after binding of the envelope trimer to the CD4 receptor (37, 38). Importantly, interaction of the HIV-1 envelope glycoprotein with CD4 in the same cell exposes these epitopes, rendering the infected cells targets for ADCC-mediating antibodies (37, 38). As with other retroviruses, interaction of the HIV-1 envelope glycoprotein with CD4 contributes to cell surface CD4 downregulation (39). Furthermore, two accessory proteins of HIV-1, Nef and Vpu, also contribute to cell surface CD4 downregulation, and Vpu also antagonizes Tetherin, which normally inhibits retroviral release (37, 38). These actions of Nef and Vpu reduce the exposure epitopes targeted by ADCC-mediating antibodies and, consequently, the susceptibility of HIV-1-infected cells to ADCC (37, 38). Thus, at least in HIV-1 infection, downregulation of the cellular receptor in the infected cell is a retroviral immune evasion strategy.

In the case of retroviruses and perhaps of other viruses, too, interaction with the cellular receptor in the producer cell may be promoted in order to confer resistance to superinfection (5). Thus, opposing selection forces may fine-tune the strength of the association between the SU and the TM subunits. This association needs to be strong enough to withstand the interaction with the cellular receptor in the producer cell (which is necessary for resistance to superinfection), without compromising incorporation of the envelope glycoprotein in the virion or causing the premature exposure of the fusion peptide in the producer cell. Equally, it must allow the conformational changes of the TM during entry and membrane fusion. It is likely that the E14 and A20 residues influence the strength of the association between the SU and the TM subunits and their mutation disrupts this fine-tuning. This notion is supported by the observation that overexpression of the mCAT1 cellular receptor also affects the infectivity of wild-type F-MLV, highlighting the interconnection of the three components, mCAT1, SU, and TM. Further elucidation of this interaction and the role of the E14 and A20 residues will also facilitate understanding of the mechanism by which immunosuppression might be mediated.

## MATERIALS AND METHODS

### Plasmids.

The E561R and A567F double mutation in the F-MLV envelope (E14R and A20F in the ISD) was introduced by mutagenesis of the respective codons (GAG → CGC and GCC → TTT, respectively) in plasmid pLRB302, containing the complete NB-tropic F-MLV clone FB29, resulting in the provirus FB29-DM. The envelope open reading frame in FB29 and FB29-DM was separately replaced with that of F-MLV clone e57, carrying either the wild-type sequence or the E561R and A567F double mutation, resulting in plasmids containing the FB29e57 and FB29e57-DM proviruses, respectively. Mutagenesis, gene synthesis, and cloning were performed by Genewiz LLC, and the constructs were verified by sequencing.

### Cell lines, infections, transfections, and transductions.

Mus dunni fibroblast cells (M. dunni cells; CRL-2017), NIH 3T3 cells (N-3T3 cells; CRL-1658), and BALB/c mouse 3T3 cells (B-3T3 cells; CCL-163) were grown in Iscove's modified Dulbecco's medium (IMDM; Sigma-Aldrich) supplemented with 5% fetal bovine serum (Gibco, Life Technologies), 2 mM l-glutamine, 100 U penicillin, and 0.1 mg/ml streptomycin. 293T cells (CRL-3216) and 293-mCAT1 cells stably transfected with murine *Slc7a1* (encoding murine cationic amino acid transporter-1 [mCAT1]) (33) were also grown in the same medium. JAWSII murine immature dendritic cells (CRL-11904) were grown in the same medium, which was additionally supplemented with 20 ng/ml recombinant granulocyte-macrophage colony-stimulating factor (Peprotech Ltd.). Cells were transfected with plasmids containing the indicated F-MLV provirus using the GeneJuice transfection reagent (Novagen) following the manufacturer's instructions. Culture supernatants with viral stocks were prepared, and the amount of virus was normalized according to the number of viral RNA copies per milliliter of supernatant, assessed by real-time quantitative reverse transcription-based PCR (qRT-PCR). Infections and transductions were carried out by adding serial dilutions of the viral stocks to target cells in the presence of Polybrene (4 μg/ml). JAWSII cell sublines, each of which carried an FB29 or FB29-DM clone, were established by single-cell sorting, performed on a FACSAria Fusion flow cytometer (BD Biosciences), of cells from JAWSII cell cultures that had been infected 2 weeks earlier with either FB29 or FB29-DM viruses that had been produced by transfection of 293T cells.

### Mice and *ex vivo* and *in vivo* infection.

C57BL/6 (B6)-backcrossed Rag1-deficient mice (40) were crossed to B6-congenic mice lacking *Emv2* (41) and additionally to IL-15 receptor α chain-deficient mice (42), creating triple mutant *Rag1*^−/−^ *Il15ra*^−/−^ *Emv2*^−/−^ mice, which lack mature T and B cells, NK cells, and ecotropic MLVs. Mice (age, 8 to 12 weeks) received an inoculum of ∼10^4^ infectious units of F-MLV clone FB29 or FB29-DM that had been produced by transfection of M. dunni cells. All animal experiments were approved by the ethical committee of The Francis Crick Institute and conducted according to local guidelines and UK Home Office regulations under the Animals Scientific Procedures Act 1986 (ASPA) (Granted Project License PPL 7007643). For *ex vivo* infection of primary B cells, spleen cell suspensions from B6 mice were stimulated with 100 μg/ml of lipopolysaccharide (LPS; Sigma-Aldrich) 48 h prior to infection with FB29 or FB29DM viruses.

### Flow cytometry.

F-MLV-infected cells were detected in suspensions of cells from cultured cell lines by flow cytometry using surface staining for the glycosylated product of the F-MLV *gag* gene, glyco-Gag (gPr80), with matrix (MA)-specific monoclonal antibody 34 (mouse IgG2b) (43), followed by an anti-mouse IgG2b-fluorescein isothiocyanate (FITC) secondary reagent (BD Biosciences), or for F-MLV SU (gp70) with the gp70-specific monoclonal antibody 720 (mouse IgG1) (43), followed by an anti-mouse IgG1-FITC secondary reagent (BD Biosciences). Cell suspensions from the spleens of infected mice were additionally stained with antibodies directly conjugated to Ter119 (eBioscience) to identify erythroid precursors. Samples were acquired with an LSRFortessa X20 flow cytometer (BD Biosciences) and analyzed with FlowJo (v10) software (Tree Star Inc.).

### Sequencing.

The entire *env* open reading frame of FB29 and FB29-DM clones isolated from JAWSII cell sublines or from chronically infected M. dunni cells was first amplified by PCR, using Ranger DNA polymerase (Bioline) and the following primers (Eurofin Genomics): forward primer 5′-ACACCAGGATTGAGCCACCA-3′ and reverse primer 5′-CCCCCTTTTTCTGGAAACTA-3′.

The amplicons were then subjected to sequencing at Source BioScience (Cambridge, UK). Sequence analyses, comparisons, and alignments were performed with the Vector NTI (v11.5) program (Invitrogen).

### Nucleic acid quantitation and expression analyses.

The number of copies of F-MLV genomic RNA in the supernatants of virus-producing cells was determined by qRT-PCR using the following primers specific for the F-MLV *env* (Eurogentec): forward primer 5′-AAGTCTCCCCCCGCCTCTA-3′ and reverse primer 5′-AGTGCCTGGTAAGCTCCCTGT-3′. The same primers were also used to quantify the expression of F-MLV *env* in RNA isolated from producer cells. The expression levels of F-MLV *env* in virus-producing murine cells were normalized by expression of *Hprt* mRNA, which was amplified with the following primers: forward primer 5′-TTGTATACCTAATCATTATGCCGAG-3′ and reverse primer 5′-CATCTCGAGCAAGTCTTTCA-3′. The expression levels of murine *Slc7a1* mRNA (encoding mCAT1) were quantified by qRT-PCR using the following primers: forward primer 5′-AGGGGCAGCGACCTGCTTTT-3′ and reverse primer 5′-CACGATGCCCACAGGAATGG-3′. These were also normalized to the level of expression of *Hprt* mRNA in murine cells and *HPRT* mRNA in human cells. The expression levels of human *HPRT* mRNA were quantified by qRT-PCR using the following primers: forward 5′-TGACACTGGCAAAACAATGCA-3′ and reverse 5′-GGTCCTTTTCACCAGCAAGCT-3′. RNA was isolated using an automated QIAcube workstation (Qiagen) and subsequently used for cDNA synthesis with a high-capacity reverse transcription kit (Applied Biosystems).

### Western blotting.

For preparation of whole-cell lysates, confluent cultures of M. dunni cells chronically infected with FB29 or FB29-DM were washed once with phosphate-buffered saline and lysed on ice with radioimmunoprecipitation assay lysis buffer (50 mM Tris-HCl, pH 7.4, 100 mM NaCl, 1% Igepal CA-630, 0.1% SDS, 0.5% sodium deoxycholate, 4 mM dithiothreitol) supplemented with cOmplete Ultra protease inhibitor cocktail (Sigma-Aldrich). Cellular debris was removed by centrifugation at 20,000 × *g* at 4°C for 10 min. The protein concentration in the supernatants was measured with a bicinchoninic acid protein assay kit (Pierce) following the manufacturer's instructions. Virions from the culture supernatants were distinguished from soluble proteins by differential centrifugation. Briefly, virions were pelleted by centrifugation at 20,000 × *g* for 2 h at 4°C. Such low centrifugal forces precipitate viral particles but not soluble proteins, and the pelleted material is referred to here as virions. Virion and soluble envelope proteins were similarly pelleted by centrifugation of the culture supernatants at a higher centrifugal force of 126,000 × *g* for 2 h at 4°C. All samples were resuspended in Laemmli sample buffer (Bio-Rad) supplemented with 10% β-mercaptoethanol and denatured by heating at 97.5°C for 5 min before loading onto a 4 to 20% mini-Protean TGX gel (Bio-Rad). Samples were then resolved by SDS-PAGE and transferred to an Immun-Blot low-fluorescence polyvinylidene difluoride membrane (Bio-Rad). The membrane was incubated for 1 h at room temperature in Odyssey blocking buffer (Li-Cor Biosciences UK Ltd.). The F-MLV envelope was detected using F-MLV SU (gp70)-specific monoclonal antibody 720 (mouse IgG1). MLV Pr65 Gag products were detected using MLV p30-specific monoclonal antibody R187 (rat IgG1) (44). Detection with primary antibodies was followed by detection with secondary goat anti-mouse IgG1 (γ1 chain specific) IRDye 680LT and goat anti-rat IgG (H+L) IRDye 800CW antibodies, respectively (both from Li-Cor Biosciences UK Ltd.). Membranes were imaged with optimized brightness and contrast by use of a Li-Cor Odyssey imaging system and further analyzed with Win Image Studio Lite (v5.2.5) software.

### Statistical analyses.

Statistical comparisons and regressions were made using SigmaPlot (v13.0) software (Systat Software Inc., Germany). Parametric comparisons of normally distributed values that satisfied the variance criteria were made by unpaired Student's *t* tests. Pairwise comparisons of data sets that did not pass the variance test were compared by the nonparametric two-tailed Mann-Whitney rank sum test.
